# Natural Infection of Omicron BA.5.2 in Patients Provides Broad Immune Responses Against SARS-CoV-2

**DOI:** 10.3390/microorganisms13040746

**Published:** 2025-03-26

**Authors:** Le Li, Tang Feng, Quan Shen, Xiaoshan Shi, Zhigong Wei, Wanze Chen, Fan Yang, Yueting Zhu, Chengxin Zhang, Shuang Zhang, Qisi Zhang, Shengwei Fu, Ning Wang, Wen-xia Tian, Jiyan Liu, Longlong Si

**Affiliations:** 1 State Key Laboratory of Quantitative Synthetic Biology, Shenzhen Institute of Synthetic Biology, Shenzhen Institutes of Advanced Technology, Chinese Academy of Sciences, Shenzhen 518055, China; 2 College of Veterinary Medicine, Shanxi Agricultural University, Jinzhong 030801, China; 3 Department of Biotherapy, Cancer Center, West China Hospital, Sichuan University, Chengdu 610041, China; 4 University of Chinese Academy of Sciences, Beijing 100049, China; 5 Institute of Biomedicine and Biotechnology, Shenzhen Institute of Advanced Technology, Chinese Academy of Sciences, Shenzhen 518055, China; 6 Shenzhen Institute of Advanced Technology, Chinese Academy of Sciences, Shenzhen 518055, China

**Keywords:** SARS-CoV-2, natural infection, breakthrough infection, herd immunity, broad immune responses

## Abstract

The implementation of COVID-19 policy and the rapid development of SARS-CoV-2 vaccines in the early pandemic significantly contained numerous outbreaks and reduced the severity and mortality of COVID-19. However, the population immunity induced by existing vaccines was insufficient to prevent SARS-CoV-2 outbreaks. The host immunity induced by the wide spread of Omicron variants and its influence on emerging SARS-CoV-2 variants are attracting broad attention. In this study, a clinical data analysis of the patients indicated that pre-vaccination reduced inflammatory responses and mitigated the severity of COVID-19 cases caused by natural infection with Omicron BA.5.2. The analysis of adaptive immune responses indicated that natural infection with BA.5.2 induced robust and broad immune responses, including both humoral and T cell-mediated immune responses (IFN-γ) against highly conserved viral antigens, and provided cross-reactive neutralization against various viral variants. Collectively, we report that the natural infection with Omicron BA.5.2 induced broad cross-reactive immunity against SARS-CoV-2 variants, which suggests that the development of a live attenuated SARS-CoV-2 vaccine with desired safety, high efficacy, broad spectrum, and long-term immune persistence is feasible. Therefore, we suggest that herd immunity, achieved through vaccination with attenuated vaccines, combined with booster doses of existing vaccines and antiviral therapy for people with high viral loads, may contribute to the eradication of this virus.

## 1. Introduction

The coronavirus disease 2019 (COVID-19) pandemic, caused by the severe acute respiratory syndrome coronavirus 2 (SARS-CoV-2), has led to public health crises worldwide. Since its outbreak at the end of 2019, China had developed multiple nonpharmaceutical intervention measures to limit the spread of SARS-CoV-2, resulting in a low infection rate in the population, which provided the country with the time to develop vaccines and immunize the population against SARS-CoV-2. Among the existing SARS-CoV-2 vaccines, the inactivated vaccine is one of the most widely used and technologically developed vaccines in China. Over 91% of the population in China have received a full primary COVID-19 vaccination schedule, and more than 53% of the vaccinated population have received a booster shot [1,2]. However, the population immunity induced by vaccines could be insufficient to prevent a SARS-CoV-2 outbreak. SARS-CoV-2 has continued to evolve, generating new variants that have led to new waves of infection and immune escape in the vaccinated population. Omicron variants have become the globally dominant circulating variants since November 2021. Up to 30 mutations was found in the spike (S) gene of Omicron variants, with half located in the receptor-binding domain (RBD) of S protein [2]. These mutations contribute to the immune escape of Omicron variants from immunity induced by existing vaccines. With the gradual relaxation of the “zero COVID” policy [3], China grappled with its largest wave of SARS-CoV-2 infections from December 2022 to January 2023, with Omicron BA.5.2 and BF.7 being the dominant variants [4]. The host immunity of individuals with natural infection of these Omicron variants and its influence on emerging variants are topics of increasing interest.

While posing threats to human health and bringing side effects, the wide infection of Omicron variants could provide herd immunity comparable to those achieved by live vaccines. Compared with existing vaccines, such as inactivated vaccines, recombinant subunit vaccines, and mRNA vaccines, live vaccines usually have significant advantages in terms of immune efficacy: a live vaccine can induce stronger and broader immune responses, especially those against viral conserved antigen epitopes, thus providing broad-spectrum cross-reactive protection against mutated viral strains. Based on this, we hypothesized that the natural infection of Omicron variants may induce immune responses against conserved antigens of SARS-CoV-2, potentially protecting populations from re-infection with other mutated SARS-CoV-2 variants and mitigating the impact of future SARS-CoV-2 waves. To test our hypothesis, we evaluated the immune responses in patients infected with Omicron BA.5.2.

## 2. Statistical Analysis

This is a retrospective study, and statistical methods were not used to determine the sample size in advance. The sample was collected based on availability, with no data excluded from the analysis. All experiments were repeated at least two times in this study. All data are presented as means ± SD. All statistical analyses were performed using GraphPad Prism software version 9.0 (GraphPad Software, San Diego, CA, USA). Comparisons of two groups were analyzed by two-tailed Student’s *t*-test. *p* values less than 0.05 (*p* < 0.05) were considered statistically significant. *p* values that were statistically significant are labeled in the Results and Figures of our manuscript. The detailed experimental materials and methods are provided in the Supplementary Materials.

## 3. Results

### 3.1. Clinical Symptoms and Inflammatory Response Markers in Patients

**Figure 1:**
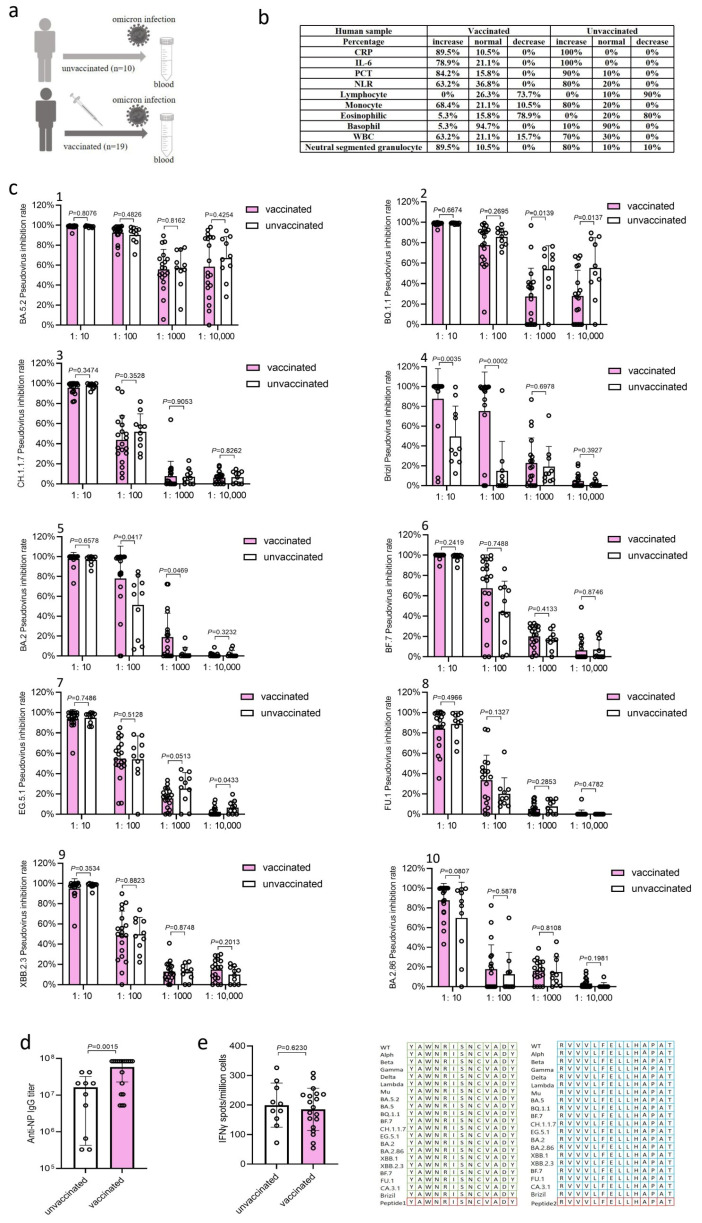
Immune characteristics of patients infected with Omicron BA.5.2. (**a**) Diagram showing the number of unvaccinated and vaccinated individuals before Omicron BA.5.2 infection. (**b**) Blood testing for indicators of inflammatory responses (C-reactive protein (CRP), interleukin-6 (IL-6), and procalcitonin (PCT)), indicators of immune imbalance (absolute lymphocyte value and neutrophil-lymphocyte ratio (NLR)), monocyte, eosinophilic, basophil, white blood cell (WBC), and neutral segmented granulocyte in vaccinated and unvaccinated patients after infection with Omicron BA.5.2. The percentages of population with normal, decreased, or increased levels of responses are shown in the table. (**c**) Neutralization of Omicron BA.5.2, BQ.1.1, CH.1.1.7, EG.5.1, BA.2, BA.2.86, XBB.2.3, BF.7, FU.1 and Gamma Brizil pseudoviruses by different dilutions of vaccinated and unvaccinated patient serum after Omicron BA.5.2 infection. (**d**) Graph showing titers of IgG antibody against NP protein of Omicron BQ.1.1 in serum of vaccinated and unvaccinated patients after infection with Omicron BA.5.2, as measured by ELISA. (**e**) Specific T cell responses against completely conserved antigen peptides of SARS-CoV-2 spike (S) protein, as measured by ELISpot assay. Two S peptides, YAWNRKRISNCVADY and RVVVLSFELLHAPAT, were used as the stimuli. The two antigen peptides were completely conserved among all SARS-CoV-2 variants. Data are presented as means ± SD. Statistical analysis was performed using an unpaired two-tailed Student’s *t*-test. *p* values less than 0.05 were considered statistically significant.

A total of 29 blood samples were collected from BA.5.2-infected patients at West China Hospital, Sichuan University, Chengdu. Among these patients, 10 had never received SARS-CoV-2 vaccine and the other 19 had received one, two, or three doses of inactivated SARS-CoV-2 vaccine prior to their BA.5.2 infection (Figure 1a). The study cohort included 16 male and 13 female patients aged 37 to 95 years. Blood samples were collected within 15 days after testing positive for SARS-CoV-2 virions. All 29 patients exhibited clinical symptoms: 18 had normal symptoms and 11 had severe symptoms.

The analysis of inflammatory response markers showed that 89.5%, 78.9%, and 84.2% of vaccinated patients experienced increases in C-reactive protein (CRP), interleukin 6 (IL-6), and Procalcitonin (PCT), respectively, which were less than those of unvaccinated patients (100% for CRP, 100% for IL-6, and 90% for PCT) (Figure 1b). During hospitalization, 73.7% of vaccinated patients and 90% of unvaccinated patients showed reductions in peripheral blood lymphocytes, an indicator of COVID-19 disease severity [5,6,7,8]. Moreover, 63.2% of vaccinated patients and 80% of unvaccinated patients showed an increase in neutrophil lymphocyte ratio (NLR), an early warning signal of severe COVID-19 [9]; 68.4% of vaccinated patients and 80% of unvaccinated patients showed an increase in monocytes; 78.9% of vaccinated patients and 80% of unvaccinated patients showed a reduction in eosinophilic, an indicator of COVID-19 disease severity; 5.3% of vaccinated patients and 10% of unvaccinated patients showed an increase in basophils; 63.2% of vaccinated patients and 70% of unvaccinated patients showed an increase in white blood cells; and 89.5% of vaccinated patients and 80% of unvaccinated patients showed an increase in neutral segmented granulocytes. Overall, the pre-vaccination reduced inflammatory responses and the severity of COVID-19 cases caused by natural infection of Omicron BA.5.2.

### 3.2. BA.5.2 Infection Induced Robust Neutralization Antibody Responses

We next tested the adaptive immune responses in patients infected with Omicron BA.5.2. We conducted a human immunodeficiency virus (HIV) pseudovirus-based neutralization assay to measure the levels of neutralization antibody in the serum samples against the original SARS-CoV-2 strain BA.5.2, Omicron variants BQ.1.1, CH.1.1.7, EG.5.1, BA.2, BA.2.86, XBB.2.3, BF.7, FU.1, and Gamma variant Brizil. The natural infection of BA.5.2 induced robust neutralization antibody responses against all tested variants in both vaccinated and unvaccinated patients (Figure 1c). The levels of anti-BA.5.2, anti-CH.1.1.7, anti-BF.7, anti-FU.1, anti-XBB.2.3 and anti-BA.2.86 neutralization antibody in the serum samples from unvaccinated patients were similar to those from vaccinated patients at dilutions of 1:10^1^, 1:10^2^, 1:10^3^, and 1:10^4^ (Figure 1c(1,3,6,8–10)). However, the levels of anti-BQ.1.1 neutralization antibody in the serum samples from unvaccinated patients were significantly (*p* = 0.0139 and *p* = 0.0137) higher compared to those from vaccinated patients at dilutions of 1:10^3^ and 1:10^4^ (Figure 1c(2)), consistent with previous reports that repeated administration of inactivated SARS-CoV-2 vaccine in breakthrough infection can inhibit the antibody response against new Omicron variants [4]. Meanwhile, the levels of anti-Brizil neutralization antibody in the serum samples from vaccinated patients were significantly (*p* = 0.0035 and *p* = 0.0002) higher than those from unvaccinated patients at dilutions of 1:10 and 1:10^2^ (Figure 1c(4)), the levels of anti-BA.2 neutralization antibody in the serum samples from vaccinated patients were significantly (*p* = 0.0417 and *p* = 0.0469) higher than those from unvaccinated patients at dilutions of 1:10^2^ and 1:10^3^ (Figure 1c(5)), and the levels of anti-EG.5.1 neutralization antibody in the serum samples from unvaccinated patients were significantly (*p* = 0.0433) higher than those from vaccinated patients at a dilution of 1:10^4^ (Figure 1c(7)).

### 3.3. BA.5.2 Infection Induced High Levels of IgG Antibody Responses

We further elucidated the immune responses against the conserved antigens of SARS-CoV-2, which are critical for cross-reactive immunity against different viral variants. The detection of IgG antibody responses in the serum of patients by an enzyme-linked immunosorbent assay (ELISA) revealed that the natural infection of BA.5.2 induced high levels of IgG antibody responses against the highly conserved SARS-CoV-2 nucleoprotein (NP) in both vaccinated and unvaccinated patients (Figure 1d), with vaccinated patients showing 3.6-fold higher antibody levels than those in the unvaccinated patients (*p* = 0.0015), suggesting that vaccination enhances the humoral immune responses induced by infection of BA.5.2 in humans.

### 3.4. BA.5.2 Infection Induced T Cell Immune Responses (IFN-γ)

Peripheral blood mononuclear cells (PBMC) were isolated from the blood samples and subjected to an enzyme-linked immunospot (ELISpot) assay to detect viral antigen-specific T cell immune responses (IFN-γ). The stimuli in the ELISpot assay were two antigen epitope peptides on S protein, YAWNRKRISNCVADY and RVVVLSFELLHAPAT, that were completely conserved among all SARS-CoV-2 variants, including wild-type (WT), Alpha, Beta, Gamma, Delta, Lambda, Mu, Omicron BA.5, Omicron BA.5.2, Omicron BQ.1.1, Omicron CH.1.1.7, Omicron EG.5.1, Omicron BA.2, Omicron BA.2.86, Omicron XBB.1, Omicron XBB.2.3, Omicron BF.7, Omicron FU.1, Omicron CA.3.1, and Gamma Brizil (Figure 1e). It was found that the natural infection of BA.5.2 induced robust T cell immune responses against the conserved antigen peptides in both vaccinated and unvaccinated patients (Figure 1e). Vaccination did not enhance the induction of T cell immune responses as evidenced by the similar levels of T cell immune responses detected in vaccinated and unvaccinated patients (Figure 1e), which is consistent with the fact that an inactivated vaccine is not able to induce T cell immune responses [10]. Overall, the natural infection of BA.5.2 induced robust and broad immune responses, including the humoral and T cellular immune responses against viral highly conserved antigens, and provided cross-reactive neutralization against different viral variants.

## 4. Discussion

The implementation of COVID-19 policy and the rapid development of SARS-CoV-2 vaccines in the early pandemic significantly contained numerous outbreaks and reduced the severity and mortality of COVID-19. In this study, the analysis of inflammatory markers (CRP, IL-6, PCT), an early warning signal of severe COVID-19 (NLR), an indicator of disease severity (eosinophils), and monocyte data in patients was performed. The data revealed that pre-vaccination helped reduce inflammatory responses and mitigated the severity of COVID-19 cases caused by natural infection with the Omicron BA.5.2 variant. Florian et al. conducted a comprehensive analysis of the clinical characteristics and immune profile changes in patients with breakthrough Delta-variants-of-concern infections after receiving one or two doses of the inactivated vaccine [11]. Their results showed that despite high levels of viral replication, two doses of the vaccine could protect the lungs from the Delta virus attack and reduce the inflammatory response caused by the virus [11]. Similarly, a study of 430 COVID-19 cases found that the ICU admission rate for patients who had received a booster dose of the inactivated vaccine was only 0.6%, significantly lower than 4.8% for those who had received two doses and 37.5% for unvaccinated patients. Additionally, patients who had received the booster dose also had shorter hospital stays and faster recovery times. The COVID-19 inactivated vaccine demonstrated protective effects in reducing severe illness and death caused by the Omicron BA.1 variant [12]. These findings are consistent with the clinical data analysis of COVID-19 patients in our study. Havervall et al. found that compared to patients who were unvaccinated or had not received a vaccine within 30–90 days after infection, those who were fully vaccinated or had received a booster dose had a lower risk of experiencing adverse health outcomes, including major cardiovascular diseases and all-cause mortality [13]. These data suggest that inactivated immunization vaccines are essential for the prevention and control of COVID-19.

Yu et al. systematically described the impact of inactivated COVID-19 vaccination on the immune response in Omicron-infected individuals, revealing the molecular mechanisms by which three booster doses of the inactivated vaccine induce the activation and maturation of monocytes, thereby exerting a potent antiviral effect [14]. Although inactivated vaccines have helped reduce mortality and clinical symptoms to some extent, studies have shown that repeated vaccination with inactivated SARS-CoV-2 vaccines dampens the production of neutralizing antibodies against Omicron variants in breakthrough infection [1]. Our study also supports this result, as the levels of anti-BQ.1.1 neutralization antibody in the serum samples from unvaccinated patients were significantly higher compared to those from vaccinated patients at dilutions of 1:10^3^ and 1:10^4^ (Figure 1c(2)). In conclusion, compared to unvaccinated individuals, those who experience breakthrough infections after vaccination typically clear the virus more quickly and have milder symptoms. Vaccination, previous infections, and hybrid immunity all provide protection against severe disease [15].

In our study, natural infection with BA.5.2 induced robust neutralization antibody responses, whereas vaccination did not enhance the induction of T cell immune responses, as evidenced by the similar levels of T cell immune responses detected in vaccinated and unvaccinated patients (Figure 1e), consistent with the fact that an inactivated vaccine is not able to induce T cell immune responses. Wang et al. revealed the long-term levels of antibodies and health status in COVID-19 convalescents following natural infection with SARS-CoV-2 and vaccination with inactivated vaccines, demonstrating that vaccination enhanced the neutralizing effect and memory B cell response against highly mutated SARS-CoV-2 variants in convalescents [16]; these further support our findings. However, studies have found that subjects with co-morbidities can have a weaker immune response [17]. Meanwhile, in some cases, natural infections do not lead to the production of neutralizing antibodies [18], which may be due to individual differences, such as differences in immune system deficiencies or insensitivity.

Given the advantages of live attenuated vaccines in terms of strength, breadth, and durability of immune responses, herd immunity achieved by the wave of Omicron variants’ infections could provide broad-spectrum protection against new variants and reduce the risk and severity of the next wave of infections. In a retrospective study of almost 25,000 patients, vaccinated individuals (who received two doses of Pfizer BNT162b2) had a 13.06-fold increased risk of breakthrough infection compared to those who had been naturally infected [19]. These results suggests that immunity due to natural infection with SARS-CoV-2 outperforms that induced by vaccination (two doses of Pfizer BNT162b2). However, recent studies have found that natural infection prior to Omicron provides strong and lasting protection against reinfection, with little decline over time. By the Omicron era, this protection was only strong in those recently infected, but it declined rapidly and significantly within a year [20]. This suggests that over time, the continuous emergence of new variants has led to immune evasion, making it challenging for existing control strategies to fully prevent reinfection. A promising solution to efficiently reduce the transmission of the variants is the development of universal live attenuated vaccines that can elicit robust and broad immunity, including humoral, T cellular, and mucosal immune responses. Currently, various live attenuated SARS-CoV-2 vaccines, including those based on gene deletion [21,22,23,24,25], genome recoding [26,27,28], codon deoptimization [29,30], and cold adaptation [31], are under development and have shown promising results.

Our data suggest that the natural infection of Omicron BA.5.2 induced neutralization antibodies against both the original strain and mutated variants, as well as IgG antibodies against the highly conserved viral NP protein, and T cellular responses against completely conserved viral antigen peptides of S protein. Although the evaluation of respiratory mucosal IgA antibodies against SARS-CoV-2 was hindered by the technical difficulty in harvesting respiratory samples, experiences from live attenuated influenza vaccines suggest that the natural infection of Omicron BA.5.2 should be able to induce respiratory mucosal IgA antibodies, which are the first defense line of adaptive immunity [11,12,13]. Therefore, some reports also suggest that the herd immunity formed by vaccinating with attenuated vaccines, combined with booster doses of existing vaccines and antiviral therapy for people with high viral loads, may help in eradicating this virus [32,33]. This aligns with our perspective on future control strategies.

In this study, we compared the clinical symptoms, inflammatory response markers, and adaptive immune responses between vaccinated and unvaccinated patients naturally infected with Omicron BA.5.2. Several points have been clarified: 1 Inactivated vaccines help prevent severe disease and inflammatory responses in patients. 2 Natural infection with Omicron BA.5.2 can induce a robust and broad immune response, which provides cross-protection against different SARS-CoV-2 variants. 3 The use of attenuated vaccines is feasible, provided that a safe and effective attenuation strategy is chosen. 4 Herd immunity induced by attenuated vaccines and targeted individual treatments contribute to the eradication of the virus. In addition, pseudovirus-based (10 types) neutralization assays were performed to measure the levels of neutralizing antibodies, and ELISpot assays were conducted to detect viral antigen-specific T cell immune responses (IFN-γ). These are the key highlights of our research. Meanwhile, our study has several limitations: 1 This is a retrospective study, and statistical methods were not used to determine the sample size in advance; a larger sample size would make our study more compelling. 2 Due to objective reasons related to sample collection, we did not divide the population into two groups based on whether they received one dose or a booster dose of the vaccine. 3 The antibody levels required to prevent infection, the inflammatory response, and severe symptoms still require further investigation.

## 5. Conclusions

Collectively, we report that the natural infection with Omicron BA.5.2 induces broad cross-reactive immunity against SARS-CoV-2 variants, which could reduce the risk of the next wave of SARS-CoV-2 infections. However, as time has passed, the evolution of the SARS-CoV-2 virus has enhanced its ability to evade immune responses. Therefore, we believe that applying existing attenuation strategies to the design of a live attenuated SARS-CoV-2 vaccine with desired safety, high efficacy, broad spectrum, and long-term immune persistence, combined with booster doses of existing vaccines and antiviral therapy for people with high viral loads, may help in eradicating SARS-CoV-2 virus.

## Data Availability

The original contributions presented in this study are included in the article/Supplementary Material. Further inquiries can be directed to the corresponding authors.

## Supplementary Materials

The following supporting information can be downloaded at: https://www.mdpi.com/article/10.3390/microorganisms13040746/s1. References [34,35,36,37,38,39,40,41] are cited in the supplementary materials.
