# Supplementary material for: Natural Infection of Omicron BA.5.2 in Patients Provides Broad Immune Responses Against SARS-CoV-2
Source: Microorganisms. 2025 Mar 26;13(4):746. doi: 10.3390/microorganisms13040746 (PMC12029644; doi:10.3390/microorganisms13040746)
Supplement: Supplementary file 1 [file microorganisms-13-00746-s001.zip › microorganisms-3555736-supplementary.pdf]

Supplementary information for

Natural Infection of Omicron BA.5.2 in Patients Provides Broad Immune Responses Against SARS-CoV-2

Le Li [1,2,†], Tang Feng [3,†], Quan Shen [1,4], Xiaoshan Shi [1,4], Zhigong Wei [3], Wanze Chen [1,4], Fan Yang [4,5], Yueting Zhu [3], Chengxin Zhang [1], Shuang Zhang [3], Qisi Zhang [1], Shengwei Fu [1], Ning Wang [6], Wen-xia Tian [2], Jiyan Liu [3,*] and Longlong Si [1,4,*]

[1] State Key Laboratory of Quantitative Synthetic Biology, Shenzhen Institute of Synthetic Biology, Shenzhen Institutes of Advanced Technology, Chinese Academy of Sciences, Shenzhen 518055, China
[2] College of Veterinary Medicine, Shanxi Agricultural University, Jinzhong 030801, China
[3] Department of Biotherapy, Cancer Center, West China Hospital, Sichuan University, Chengdu 610041, China
[4] University of Chinese Academy of Sciences, Beijing 100049, China
[5] Institute of Biomedicine and Biotechnology, Shenzhen Institute of Advanced Technology, Chinese Academy of Sciences, Shenzhen 518055, China
[6] Shenzhen Institute of Advanced Technology, Chinese Academy of Sciences, Shenzhen 518055, China

* Correspondence: liujiyan1972@163.com (J.L.); ll.si@siat.ac.cn (L.S.)
† These authors contributed equally to this work.

Materials and Methods

Human samples

Blood samples were collected from 26 individuals infected with the Omicron variant (BA.5.2) during an epidemic from December 2022 to February 2023 in Chengdu, China. According to the 10th edition of National COVID-19 Guidelines, the disease is classified into mild, normal, severe and critical types. The vaccination history of hospitalized patients was recorded and the patients were divided into two groups: vaccinated (Sinovac Biotech or Chengdu Bio) and unvaccinated.

Pseudovirus production and neutralization assay

The neutralization capacity of serum from vaccinated and unvaccinated patients was measured by HIV-based BA.5.2 and BQ.1.1 pseudovirus assay.

Genes coding for Omicron BA.5.2 S protein (GISAID No. EPI_ISL_16404950), BQ.1.1 S protein (GISAID No. EPI_ISL_15542649), CH.1.1.7 S protein (GISAID No. EPI_ISL_15641263), EG.5.1 S protein (GISAID No. EPI_ISL_17308785), BA.2 S protein (GISAID No. EPI_ISL_6795834), BA.2.86 S protein (GISAID No. EPI_ISL_18096761), XBB.2.3 S protein (GISAID No. EPI_ISL_16311589), BF.7 S protein (GISAID No. EPI_ISL_12810243), FU.1 S protein (GISAID No. EPI_ISL_17012469) and Gamma Brazil (B.1) S protein (GISAID No. EPI_ISL_412964) were cloned into pcDNA3.1 plasmid, generating pcDNA3.1-BA.5.2 S, pcDNA3.1-BQ.1.1 S, pcDNA3.1-CH.1.1.7 S, pcDNA3.1-EG.5.1 S, pcDNA3.1-BA.2 S, pcDNA3.1-BA.2.86 S, pcDNA3.1-XBB.2.3 S, pcDNA3.1-BF.7 S, pcDNA3.1-FU.1 S and pcDNA3.1-Brazil S respectively. To generate pseudovirus, HEK293T cells were seeded into 10-cm dishes and cultured for 24 h. When the confluency reached 80%, the cells were co-transfected with 10 μg NL4-3.Luc.R-E plasmid and 10 μg pcDNA3.1-BA.5.2 S, pcDNA3.1-BQ.1.1 S, pcDNA3.1-CH.1.1.7 S, pcDNA3.1-EG.5.1 S, pcDNA3.1-BA.2 S, pcDNA3.1-BA.2.86 S, pcDNA3.1-XBB.2.3 S, pcDNA3.1-BF.7 S, pcDNA3.1-FU.1 S or pcDNA3.1-Brazil S using a ProFection® Mammalian Transfection System (Promega, Cat# E1200), according to the manufacturer's instructions. 8 hours after transfection, the transfection medium was changed to DMEM (Gibco, Cat# C11995500BT) supplemented with 5% FBS (PAN, Cat# ST30-3302), 1% penicillin, and 1% streptomycin (GENOM, Cat# GNM15140-1). The cells were cultured for another 48 hours, and pseudovirus-containing supernatants were collected.

To test the neutralization activity of patient serum, angiotensin-converting enzyme 2 (ACE2)-expressing HEK293T (HEK293T-ACE2) cells at 6×10^4 cells/well were seeded into 96-well plates (Corning, Cat#3595) in DMEM supplemented with 10% FBS (PAN, Cat# ST30-3302), 1% penicillin, and 1% streptomycin (GENOM, Cat# GNM15140-1) and cultured for 24 hours at 37 °C in 5% CO2. Serum samples were diluted (1:10, 1:100, 1:1000, 1:1000) in DMEM (Gibco, Cat# C11995500BT) supplemented with 2% FBS (PAN, Cat#

ST30-3302), 1% penicillin, and 1% streptomycin (GENOM, Cat# GNM15140-1). 50 μL of the diluted serum samples and 50 μL of pseudovirus were mixed and added to each well of the 96-well plates. The wells with only medium was used as blank control, and the wells with only pseudovirus were used as positive control. After 48 hours of incubation at 37°C in 5% CO_2, luciferase activity was determined using a Bright-Lite Luciferase Assay System assay kit (Vazyme, Cat# DD1204-01), according to the manufacturer's instructions. Briefly, 70 μL of medium supernatants were discarded from reach well of the 96-well plates and 30 μL of substrate solution was added. After 2 minutes of incubation, the mixture was transferred to black-well plates (SPL life sciences, Cat# 30296) and luminescence was detected using a multi-mode microplate reader (Bio-Tek Synergy H1). Pseudovirus inhibition rate was calculated as [1-(sample well signal-blank control well signal) / (positive control well signal-blank control well signal)] × 100. Data were analyzed using GraphPad Prism 9 (GraphPad Software, USA).

Detection of IgG against SARS-CoV-2 NP by ELISA

SARS-CoV-2 Omicro (BQ.1.1) NP protein (Sino biotech, Cat# 40588-V07E39) was diluted to 1 μg/mL in ELISA coating buffer (Solarbio, Cat# C1055). 96-well plates (Corning, Cat# 3590) were coated with 50 μL diluted NP solution at 4℃ overnight, washed three times with PBST, and blocked with PBST containing 5% skim milk (YEASEN, Cat# 36120ES60) for 1 hour at 37℃. Then blocking solution was removed and the plates were washed three times with PBST. Patient serum samples were 2-fold serially diluted starting at 1:10 in PBST containing 5% skim milk. 100 μL of the diluted serum samples were added to each well of the 96-well plates coated with NP protein and incubated at 37℃ for 2 hours. The 96-well plates were washed three times with PBST and incubated with HRP-Goat Anti-Human IgG (H+L) (Proteintech, Cat# SA00001-17 ; 1:2000 dilution) diluted in PBST containing 5% skim milk at 37°C for 1.5 hours. After washing three times with PBST, 100 μL of 3,3',5,5'-tetramethyl benzidine (TMB) substrate (Beyotime, Cat# P0209) was added to each well and

incubated for 15 minutes at room temperature, followed by the addition of 100 μL of ELISA stop solution (Solarbio, Cat# C1058). The spectroscopic absorbance of each well was read at a wavelength of 450 nm using a microplate reader (BioTek Synergy H1).

Peptides design and preparation

S proteins of the WT, Alpha, Beta, Gamma, Delta, Lambda, Mu, and Omicron variant strains (BA.5, BA.5.2, BQ.1.1, CH.1.1.7, EG.5.1, BA.2, BA.2.86, XBB.1, XBB.2.3, BF.7, FU.1 and CA.3.1), Gamma variant (Brizil) were aligned. Two completely conserved peptides (Peptide-1: YAWNRKRISNCVADY; Peptide-2: RVVVLSFELLHAPAT) were selected from S proteins according to the references[34-49]. Peptide synthesis and validation were conducted by GenScript Biotechnology Co., LTD. Peptide-1 was dissolved in 0.2M acetic acid and then diluted in RPMI 1640 medium (Gibco, Cat# C11875500BT). Peptide-2 was dissolved in DMSO and then diluted in RPMI 1640 medium (Gibco, Cat# C11875500BT). The working concentration of the peptide solution was 5 μg/mL.

IFN-γ ELISpot assay

PBMCs were isolated from heparinized whole blood using Ficoll density gradient sedimentation method (UElandy, H9003), according to the manufacturer's instructions[40-41]. SARS-CoV-2 S peptides-specific T cell responses were measured by an IFN-γ ELISpot assay (Mabtech, Cat# 3321-4AST-2), according to the manufacturer's instructions. Briefly, the pre-coated 96-well plates supplied in the kit were washed four time with PBS and added with 200 μL of RPMI 1640 medium (Gibco, Cat# C11875500) supplemented with 10% FBS (PAN, Cat# ST30-3302). After 30 minutes of incubation at room temperature, isolated PBMC suspension was added to the 96-well plates at 10^5 cells/well, followed by the addition of SARS-CoV-2 S peptide solution described above. After 24-30 hours of incubation at 37 °C in 5% CO_2, the cells were discarded and the plates were washed five times with PBS, followed by the sequential addition of detection antibody R4-6A2, streptavidin-ALP, and BCIP/NBT-plus substrate supplied in the kit. The reaction was stopped by extensive wash

with water when obvious spots emerged. The spot numbers were recorded with an ELISpot reader (Cellular Technology).

Supplementary references

34. Zhao, J.; Wang, L.; Schank, M.; Dang X.D.; Lu, Z.Y.; Cao, D.C.; Khanal, S.S.; Nguyen, L.N.; Nguyen, L.N.T.; Zhang, J.Y.; et al. SARS-CoV-2 specific memory T cell epitopes identified in COVID-19-recovered subjects. Virus Res. 2021, 304, 198508.

35. Verhagen, J.; van der Meijden, E.D.; Lang, V.; Kremer, A.E.; Volkl, S.; Mackensen, A.; Aigner, M.; Kremer, A.N. Human CD4+ T cells specific for dominant epitopes of SARS-CoV-2 Spike and Nucleocapsid proteins with therapeutic potential. Clin. Exp. Immunol. 2021, 205, 363-378.

36. Peng, Y.; Mentzer, A.J.; Liu, G.; Yao, X.; Yin, Z.X.; Dong, D.D; Dejnirattisai, W.; Rostron, T.; Supasa, P.; Liu, C.; et al. Broad and strong memory CD4+ and CD8+ T cells induced by SARS-CoV-2 in UK convalescent individuals following COVID-19. Nat. Immunol. 2020, 21, 1336-1345.

37. Low, J.S.; Vaqueirinho, D.; Mele, F.; Foglierini, M.; Jerak, J.; Perotti, M.; Jarrossay, D.; Jovic, S.; Perez, L.; Cacciatore, R.; et al. Clonal analysis of immunodominance and cross-reactivity of the CD4 T cell response to SARS-CoV-2. Science 2021, 372, 1336-1341.

38. Knierman, M.D.; Lannan, M.B.; Spindler, L.J.; McMillian, C.L.; Konrad, R.J.; Siegel, R.W. The Human Leukocyte Antigen Class II Immunopeptidome of the SARS-CoV-2 Spike Glycoprotein. Cell Rep. 2020, 33, 108454.

39. Chen, J.; Wang, P.; Yuan, L.; Zhang, L.; Zhang, L.M.; Zhao, H.; Chen, C.J.; Wang, X.J.; Han, J.L.; Chen, Y.D.; et al. A live attenuated virus-based intranasal COVID-19 vaccine provides rapid, prolonged, and broad protection against SARS-CoV-2. Sci. Bull. 2022, 67, 1372-1387.

40. Chevalier, M.F.; Bobisse, S.; Costa-Nunes, C.; Cesson, V.; Jichlinski, P.; Speiser, D.E.; Harari, A.; Coukos, G.; Romero, P.; Nardelli-Haefliger, D.; et al. High-throughput monitoring of human tumor-specific T-cell responses with large peptide pools. Oncoimmunology 2015, 4, e1029702.

41. Wang, J.; Li, K.; Mei, X.; Cao, J.P.; Zhong, J.Y.; Huang, P.Y.; Luo, Q.; Li, G.C.; Wei, R.; Zhong, N.S.; et al. SARS-CoV-2 vaccination-infection pattern imprints and diversifies T cell differentiation and neutralizing response against Omicron subvariants. Cell Discov. 2022, 8, 136.
